# Assessment of Liver Transplant Donor Biopsies for Steatosis Using Frozen Section: Accuracy and Possible Impact on Transplantation

**DOI:** 10.4021/jocmr629w

**Published:** 2011-07-26

**Authors:** Benjamin Heller, Stephen Peters

**Affiliations:** a New Jersey Medical School, University of Medicine and Dentistry of New Jersey, Newark, New Jersey, USA

## Abstract

**Background:**

Pre-transplant frozen section evaluation for macrovesicular steatosis has long been used as a guide for donor liver utility, but may not agree with the permanent section evaluation. This study sought to evaluate the accuracy of frozen section in an active transplant service.

**Methods:**

Retrospective review of cases where frozen section analysis was undertaken to assess percent macrovesicular steatosis was performed, comparing the frozen section diagnosis to the final diagnosis.

**Results:**

Ninety-six cases were available for review. In 7 of these cases (7%), the difference between the two slides was significant; that is, the difference between the two slides may have contributed to a change in clinical management at a cutoff of 30%.

**Conclusions:**

Clinicians need to be aware that accuracy is satisfactory in experienced hands but some discrepancies may occur.

**Keywords:**

Liver; Steatosis; Pathology

## Introduction

Pre-transplant histopathological evaluation of percent of macrovesicular steatosis has long been used as a guide for donor liver utility [1]. Intra-operative frozen section evaluation by pathologists has been used to accomplish this task [2, 3]. A high degree of macrovesicular steatosis is associated with poor graft function [4, 5]. Specifically, livers with macrovesicular steatosis greater than 60% have a high risk of dysfunction, compared to livers with less than 30% macrovesicular steatosis, which perform considerably better [6]. Percent steatosis cutoff values vary from institution to institution with 30% frequently used as a conservative approach [4, 7]. These values are later compared to a postoperative analysis of the same tissue. Variability in frozen section interpretation may be a result of a variety of factors. Variations in frozen section preparation technique used to embed, freeze and stain tissues may lead to differences in appearance in the final preparation. Variability in biopsy location, and pathologist subjectivity and experience are also factors which can lead to disparities in interpretation [8]. We sought to evaluate the accuracy of the frozen section interpretation in a busy transplant service.

## Materials and Methods

This was an IRB approved retrospective analysis of donor livers evaluated at University Hospital in Newark, NJ from September 2001 to June 2010. Cases were identified from pathology logs. All cases evaluated intraoperatively for macrovesicular steatosis were included. Ninety-six liver biopsies from donors were evaluated. Data collected from the reports included the percent macrovesicular steatosis as assessed on frozen section and on permanent section. When a range was given, the mean value was used for analysis. Agreement was defined as identical or overlapping values. For all values that differed, we documented if the frozen section was an over estimate or an under estimate, and the amount of the difference.

## Results

Of the 96 specimens, 63 (66%) had agreement between the frozen section diagnosis and the final diagnosis. In 33 of the cases (34%), the values between the frozen section and final diagnoses differed.

In 13 of these cases (14%), the frozen section interpretation value overestimated the amount of steatosis. In 20 of these cases (21%), the frozen section interpretation value underestimated the amount of steatosis.

**Figure 1.**
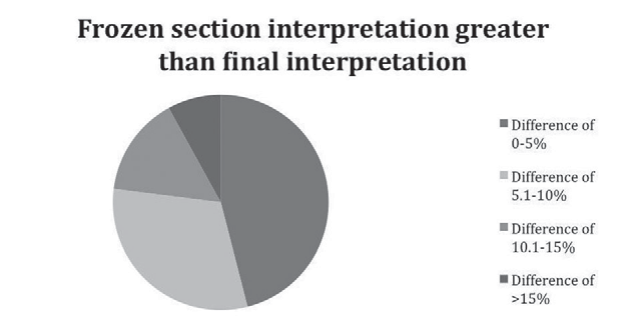
Demonstrates how much greater the deviation of the frozen section was from the permanent section.

**Figure 2.**
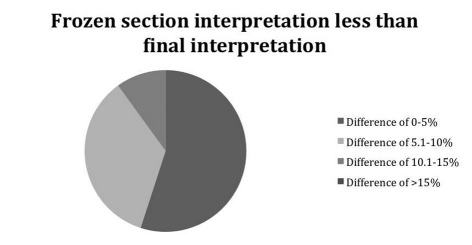
Demonstrates how less the deviation of the frozen section was from the permanent section.

In the majority of cases, the discrepancy was unlikely to be clinically significant, i.e. would not have caused a useable liver to be discarded or a suboptimal liver to be utilized. Most cases varied by less than 10% (Fig. 1, 2).

Cases were considered to have clinically significant implications if the discrepancy would have altered management, using a cutoff value of 30%. Donor livers that had a frozen section steatosis value greater than 30%, but were later found to be less than or equal to 30% occurred in 3 cases (3%). Donor livers that had a frozen section steatosis value less than 30%, but were later found to be greater than or equal to 30% occurred in 4 cases (4%) for a total of 7 cases (7%) that were considered to have clinically significant discrepancies.

## Discussion

Although frozen sections are a useful and accurate modality for the evaluation of tissue, there are known pitfalls. The ability to correctly interpret a frozen section will vary with the quality of the preparation. There can be considerable variation in the quality of frozen sections preparations depending on techniques employed and the training and experience of cryotomists.

Techniques used to embed the tissue may lead to considerable variability in the final "footprint" of the tissue to be compared in the frozen section and permanent section preparation. When embedding a core or wedge biopsy sample the first goal is to embed the tissue in a single flat plane. One needs to complete trim the block to achieve the complete tissue face on the frozen section while minimizing the tissue wastage. This will offer the best chance to achieve a similar size and shape tissue face (footprint) so for final comparison of the frozen section and permanent section preparation.

Face up embedding of a wedge biopsy which is more pyramidal and irregular in its dimension is more likely to result in preparations which no longer appear similar on comparison. Likewise a core biopsy not embedded in a flat plane with minimal trimming will often result in significant tissue wastage. The footprint will be different and likely smaller when to compare in the frozen section and permanent preparations. Optimal embedding can best be accomplished using a face down embedding technique as opposed to face up embedding. Face up embedding refers to simply freezing the tissue with the desired face up on a chuck with or without compression by a heat extractor. This can lead to variability in the flatness of the plane of the tissue as well as variation in "x-y" orientation of the preparation and potential tissue wastage. Face down embedding can be accomplished using a variety of techniques utilizing well bars, plastic molds or simply freeing the tissue first in a flat plane on any freezing temperature surface such as the cryostat stage or a heat extractor [9].

The technique used to freeze the tissue can also introduce variability in the appearance of the frozen section preparation. Techniques which freeze the tissue more slowly will introduce a greater degree of freeze artifact which can appear as clear spaces occupied by ice crystals. The clear spaces occupied by ice crystal can resemble fat vacuoles leading to misinterpretation of the percentage of cells containing macrovesicular fat. This variation can be minimized using techniques which accomplish more rapid freezing [9].

Subjectivity and pathologist experience are sources of variability. We sought to evaluate the accuracy of frozen section analysis of percent macrovesicular steatosis in assisting decision making for liver transplantation. Our institution has a busy transplantation service and hence the pathologists have experience in evaluating these specimens. This may not be the case in institutions that do not have active transplant services.

There are only a few studies that that have looked at the experience of other institutions. In a 1993 study, Markin *et al.* noted that after implementation of frozen-section analysis, primary nonfunction in recipients dropped from 8.5% to 1.4% [1]. They stated that frozen section is a reliable tool for evaluating the appropriateness of a liver for use in transplant. Fiorentino *et al.*, using a cutoff of 30%, recorded an overestimation of the amount of steatosis at frozen-section in just 1.4% of biopsies. This is markedly lower than in our study. They state that frozen-section histological analysis is an effective and predictive method for liver transplantation [3]. However, El-Badry *et al.* has argued that the frozen section is no longer the gold standard for hepatic steatosis [8]. They compared 46 cases among 4 expert pathologists from multiple institutions across Europe and the United States. The pathologists disagreed with one another, and disagreed with computerized quantification of steatosis.

When a pathologist simply "eyeballs" a slide to appraise some ratio or percentage of hepatocytes containing fat vacuoles versus those without vacuoles it is usually some form of estimate based on the pathologist's personal perception. A more objective approach would be to use a comparison chart which illustrates liver tissue with fatty change of known percentages in increments varying by 10% as defined by morphometric analysis.

Frozen-section histopathological analysis is a valuable adjunct but is not entirely accurate. Our study demonstrated that in 7% of cases, the decision to transplant might have been adversely affected by frozen section analysis. Performance of these frozen-sections in institutions where the pathologists are experienced in their interpretation, as well as meticulous technique of slide production will minimize the discrepancies. Creation of a comparison chart with morphometrically defined percentages would offer the reviewing pathologist a tool for more objective assessment of percentage of steatosis. Clinicians need to be aware that accuracy of frozen section analysis of macrovesicular steatosis in liver is good in experienced hands but some discrepancies will occur.
